# Relationships between fatigue severity scale (FSS)/ scale for mood assessment (EVEA) and clinical manifestations in spanish long-COVID patients

**DOI:** 10.1371/journal.pone.0324075

**Published:** 2025-07-07

**Authors:** Esther Bahillo Ruiz, Lucía Pérez-Pérez, Rosa M. Cárdaba-García, Carlos Durantez-Fernández, Lourdes Jiménez-Navascués, Veronica Velasco-Gonzalez, Alba Muñoz-del Caz, Miguel Madrigal, Elena Olea

**Affiliations:** 1 Nursing Care Research (GICE), Faculty of Nursing, University of Valladolid, Valladolid, Spain; 2 Nursing Department, Faculty of Health Sciences, University of Valladolid, Soria, Spain; 3 Nursing Department, Faculty of Nursing, University of Valladolid, Valladolid, Spain; 4 Primary Care Management Valladolid West (SACYL), Valladolid, Spain; 5 University Clinical Hospital of Valladolid, Valladolid, Spain; 6 Unidad de Excelencia Instituto de Biomedicina y Genética Molecular (IBGM). Universidad de Valladolid-CSIC, Valladolid, Spain; Northwestern University Feinberg School of Medicine, UNITED STATES OF AMERICA

## Abstract

**Aim:**

This study aims to investigate the influence of fatigue and mood disturbances in Spanish long-COVID patients and to establish relationships between these factors and other clinical manifestations.

**Method:**

A descriptive correlational study was conducted using a self-administered online questionnaire. The sample was obtained through non-random convenience sampling, comprising 374 participants from various regions of Spain. Data collection occurred between July 2, 2022, and November 30, 2022. The questionnaire collected demographic information and inquired about symptomatology as well as self-perception of health status. Validated scales, namely the Fatigue Severity Scale (FSS) and the Scale for Mood Assessment (EVEA), were utilized.

**Results:**

The non-random sample consisted of 374 participants from diverse regions of Spain, of whom 79.9% were women. Over 70% of participants reported fatigue, while the EVEA revealed high scores in sadness-depression (4.94 ± 2.82) and anxiety (4.57 ± 2.88). Significant relationships were identified between fatigue and mood disturbances and neurological, psychological, locomotive, and pain symptoms.

**Conclusions:**

Given the impact of the syndrome on psychological, social and economic spheres, regular monitoring of patients with long COVID is crucial. This study corroborates previous research findings and is notable for demonstrating the persistence of symptoms for over a year. Mood disorders, such as anxiety and depression, are closely related to physical symptoms, highlighting the need for holistic healthcare approaches.

## Introduction

In December 2019, the disease caused by the SARS-CoV-2 virus (severe acute respiratory syndrome coronavirus 2), called COVID-19, appeared in Wuhan, China. During acute infection with the virus patients exhibited common symptoms such as fatigue, shortness of breath, and cognitive dysfunction. Since 2019, according to the World Health Organization (WHO), more than 760 million cases and 6.9 million deaths have been recorded worldwide [1]. In Spain there have been 13,914,811 confirmed cases of COVID-19 and 121,760 deaths as of June 30, 2023 [2]. Approximately 10–20% of patients with COVID-19 have persistent sequelae, although recent studies raise that estimation to 34% of those affected [3]. These patients have multi-organ involvement with repercussions on their quality of life. No clearly established relationship has been found until now, with the severity of the acute phase of the disease [4–6]. Several studies indicate a higher prevalence of persistent symptoms among women (OR 1.5) which suggests there may be a link between gender and long-COVID syndrome [7,8].

The persistence of symptoms for more than 2 months or the appearance of new ones 3 months after the initial SARS-CoV-2 infection, with no apparent medical explanation, is defined by the WHO as Long-COVID or long COVID [9]. The National Institute for Health and Care Excellence (NICE) defines it as a set of signs and symptoms that persist for more than 12 weeks and develop during or after COVID-19 infection, with no other diagnosis to explain it [10]. The term long-COVID is the most commonly used term to refer to this post-viral syndrome of unknown cause and with characteristics similar to chronic fatigue.

The scientific literature provides evidence on the relationship between Long-COVID and quality of life in patients [11,12]. Reduced quality of life can occur in up to 37% of patients suffering from this disease [13]. The international consensus study, conducted via a comprehensive review of research on this issue, provided a classification of eleven possible symptoms of the long-COVID condition in adults. These were: fatigue, pain, post-exertion symptoms, work or occupational and study changes, survival, as well as cardiovascular, respiratory, nervous system, cognitive, mental health, and physical conditions [14]. Among these symptoms, one of the most prevalent is fatigue. The ratio of individuals who experienced fatigue for 12 weeks or more after a COVID-19 diagnosis was 0.32 (95% CI, 0.27, 0.37; p < 0.001; n = 25268; I2 = 99.1%) [15]. This fatigue manifests itself in the form of dyspnoea, difficulty concentrating, anxiety, pain, or headaches [16–18].

Anxiety-depression is another common symptom. Long-COVID patients present this problem more often than people who have suffered from the acute form of the disease but did not display persistent sequelae [19]. Although cognitive problems affect between 10 and 25% of patients who experience Long-COVID [20], these symptoms may sometimes be influenced by the consumption of a high number of drugs can affect mnesic and cognitive features, mainly due to the intake of psychotropic drugs of the benzodiazepine spectrum and some selective serotonin reuptake inhibitors (SSRIs) [21]. However, it seems to have been demonstrated that some long-COVID patients present lesions and/or alterations in the morphological substrate of the brain parenchyma, specifically in gyri of the temporal and/or frontal cortex, which may explain the neuropsychiatric symptoms associated with the syndrome [22,23].

Despite the increasing care provided to these patients, there remains a lack of systematic characterisation of the symptoms they experience, both in terms of their frequency and intensity [13]. Conducting a descriptive study is a crucial first step in understanding the magnitude and specific characteristics of fatigue and mood disturbances within this population. Fatigue appears to significantly impact patients’ quality of life due to its debilitating nature [15]. While international research is available, local social, cultural, and healthcare contexts may influence the manifestation and perception of these symptoms. Providing data on the situation of these patients in Spain is vital for designing public health policies tailored to the local context, prioritising resources, and developing specific rehabilitation and psychological support programmes [24].

The aim of this study is to determine the prevalence of long-COVID symptoms in the Spanish population belonging to an association of patients with this syndrome and to establish relationships between mood alterations and fatigue with other clinical manifestations such as pain and neurological, psychological, locomotive, respiratory, digestive, dermatological, cardiac, and renal disorders, among others.

## Methodology

### Participants and procedure

Long-COVID patient associations from all over Spain were contacted, obtaining a non-random sample of 374 volunteer participants who met the inclusion criteria of the study: patients with persistent symptoms of COVID-19 according to the WHO criteria (persistence of symptoms for more than 2 months or the appearance of new ones 3 months after the initial SARS-CoV-2 infection, with no apparent medical explanation) [9], who are members of a long-COVID association in Spain, have basic skills in the use of electronic devices, have a command of Spanish and are willing to participate in the study. Considering that some groups of long-COVID patients numbered around 800, the estimate of the sample size, with a certainty of 95% and an accuracy of 3%, would be 130 people. Assuming possible participant attrition due to incomplete responses to the questionnaire, a larger sample size was required. A loss rate of 15% was accepted, resulting in the need for an adjusted sample size of 149 individuals. The prevalence of Long COVID in Spain was estimated to be 15%. This parameter was therefore used to calculate the sample.

Data were collected through online self-administered questionnaires. The link to the questionnaire was disseminated by email to the patients in each association, using snowball sampling to achieve an adequate sample volume. A descriptive correlational investigation was carried out between mood alterations and fatigue in these patients with different clinical manifestations.

Data collection was conducted from July 2 2022 to November 30 2022. The study was approved by the Ethics Committee of the Eastern Area of Valladolid, with registration number PI-22–2747.

In accordance with the provisions of the Declaration of Helsinki, each participant received written information about the purpose of this study and gave written informed consent. They were also provided with an e-mail address in case they had any doubts.

### Variables

The self-administered questionnaire that was distributed included questions organized into 3 sections: a) demographic data and health situation related to COVID-19, b) information on the clinical situation and self-perception of health status, and c) validated scales to assess fatigue (Fatigue Severity Scale) and mood (Scale for Mood Assessment).

#### (a) Demographics and health status related to COVID-19.

The information collected in this section was as follows: Age, Gender, Employment Status, Previous Chronic Illness, Duration of COVID-19 Symptoms and if they persisted at the time of answering the questionnaire, what type of persistent symptoms participants were suffering from (specifying between neurological, psychological or emotional, locomotive, respiratory, digestive, dermatological, cardiac, renal, pain or others), whether participants had required hospitalization for COVID-19, length of admission, if participants had been admitted to the ICU, the number of times they had been infected, whether they had been vaccinated against SARS-CoV-2, and how many doses of the vaccine they had been given.

#### (b) Information on the clinical situation and self-perception of the state of health.

Dichotomous Yes/No scale: Breathlessness, Fatigue and Sleep problems. Likert scale from 1 (I have no problems in that area) to 3 (I am disabled in that area) to assess: Mobility, Personal care, Everyday activities (e.g., work, study, housework, family or leisure activities), Pain/Discomfort and Anxiety/Depression. Likert-type scale from 1 (I only feel breathless when doing intense exercise) to 5 (I feel breathless when doing everyday things such as getting dressed, leaving the house or when standing up) in the category Breathlessness and activity. Score from 0 to 10 in the health status category.

#### (c) Validated scales to assess fatigue and mood.

Fatigue Severity Scale (FSS): In 1989, Krupp, LaRocca, Muir-Nash, and Steinberg [25] created this questionnaire to assess fatigue in people with chronic illnesses. It is a 9-item Likert questionnaire, in which the answers have 7 possible values ranging from 1 (“strongly disagree”) to 7 (“strongly agree”). The mean score of the items is used as the FSS score, considering a state of “fatigue” when the FSS score is ≥ 5, and a state of “no fatigue” for scores ≤4; Scores between 4.1 and 4.9 were considered “doubtful fatigue”. During this research, the Spanish version of the questionnaire was used [26]. It should be noted that this instrument has been validated with good results for patients with COVID-19 [27]. The reliability of the instrument, measured by Cronbach’s alpha, exhibits high internal consistency, with α values ranging from 0.81 to 0.94 [28,29]. Some studies have determined the FSS score as the sum of the nine items, but this scoring system is not validated.

Scale for Mood Assessment (EVEA): instrument developed for the Spanish population in 2001 by Sanz [30], which was translated into English by the same author [31]. This questionnaire is composed of 16 items, scored using an 11-point graphical Likert scale, from 0 (not at all) to 10 (very much). It aims to assess four moods: anxiety, anger-hostility, sadness-depression, and happiness. Each mood is represented by four items with different adjectives, which define a subscale, and all items within each subscale are formulated in the same direction. The scale proposes 4 results, one for each mood studied, which are scored between 0 and 10 and are obtained by directly adding the score of the four adjectives corresponding to each subscale and dividing the sum by 4. The Spanish version achieves a reliability value of internal consistency (Cronbach’s alpha) varying between 0.86 and 0.92, with a mean of 0.88, for the sadness-depression subscale, between 0.92 and 0.94, with a mean of 0.92, for anxiety, between 0.93 and 0.95, with a mean of 0.93, for the anger-hostility subscale and between 0.88 and 0.96, with an average of 0.92, for happiness [32].

### Statistical analysis

Measures of central distribution (mean) and dispersion (standard deviation [SD]), as well as absolute frequencies and percentages, were used to describe the data. To analyse normality, the Kolmogorov-Smirnov test was used, using the Student’s t-test and ANOVA as parametric tests and Mann-Whitney and Kruskal-Wallis U tests as non-parametric tests. Pearson’s chi-square test was used for categorical variables. The analysis of the self-assessment of the variables on health status was performed using multinomial logistic regression. The tools used for the analysis were IBM SPSS Statistics version 26.0 (SPSS Inc., Chicago, IL, USA), R version 4.0.3 (The R Foundation for Statistical Computing, Vienna, Austria) and Microsoft Excel® for Microsoft 365. In all tests, a confidence level of 95% and a p-value of less than 0.05 were considered significant.

## Results

A total of 374 participants were recruited from patients belonging to long-COVID associations in various regions of Spain. Table 1 shows the analysis of their sociodemographic characteristics and their health situation in relation to COVID-19. The reliability of the instrument in our sample showed a high internal consistency, the Cronbach’s alpha in FSS was 0.96, meanwhile, in EVEA varying between 0.90 and 0.92 (EVEA Sadness-depression = 0,90, EVEA Anxiety = 0.92, EVEA Anger-hostility = 0.92 and EVEA Happiness = 0.92).

**Table 1:** Frequency distribution and percentages of general study variables.

Variables	n (%) mean ± SD	Variables	n (%) mean ± SD
**Age**	47.01 ± 9.85	**What kind of persistent symptoms do you have?**	
**Gender**		Neurological	297 (79.4)
Male	75 (20.1)	Psychological or emotional	217 (58.0)
Female	299 (79.9)	Locomotive	200 (53.5)
**Employment Status**		Respiratory	243 (65.0)
Active	149 (39.8)	Digestive	200 (53.5)
Unemployed	49 (13.1)	Dermatological	137 (36.6)
Temporary disability	154 (41.2)	Cardiac	129 (34.5)
Permanent disability	22 (5.9)	Renal	30 (8.0)
**Previous chronic illnesses**	81 (21.7)	Pain	284 (75.9)
**Do you continue having symptoms?**	360 (96.3)	Other	154 (41.2)
**How many times have you been infected?**		**Did you require hospitalisation for COVID-19?**	80 (21.4)
Once	250 (66.8)	**Time of admission**	15.01 ± 17.29
Twice	113 (30.2)	No admission	2 (0.5)
3 times	9 (2.4)	1 to 3 days	12 (3.2)
More than 3 times	2 (0.5)	4 to 7 days	18 (4.8)
**Length of COVID-19 symptoms**		8 to 14 days	20 (5.3)
Less than 1 month	9 (2.4)	More than 15 days	28 (7.5)
Between 1 and 3 months	10 (2.7)	**Did you require admission to the ICU?**	15 (4.0)
Between 3 and 6 months	32 (8.6)	**Scale for Mood Assessment (EVEA)**	
Between 6 months and 1 year	42 (11.2)	EVEA Sadness-depression	4.94 ± 2.82
More than 1 year	281 (75.1)	EVEA Anxiety	4.57 ± 2.88
**Have you been vaccinated against SARS-CoV-2?**	330 (88.2)	EVEA Anger-hostility	4.30 ± 2.90
**How many doses have you been given?**		EVEA Happiness	3.06 ± 2.32
1 dose	43 (13.0)	**Fatigue Severity Scale Mean (FSS-M)**	5.45 ± 1.82
2 doses	185 (56.1)	No fatigue (≤4)	86 (23.0)
3 doses	102 (30.9)	Doubtful fatigue (4.1–4.9)	16 (4.3)
		Fatigue (≥5)	272 (72.7)

Regarding the FSS scores, mostly of participants had high levels of fatigue. Patients with high levels of fatigue had much longer lasting symptoms, usually between 3 months and over a year (p < 0.001), and continued having symptoms (p < 0.001). High rates of incapacity for work (p < 0.001) were also observed (Table 2).

**Table 2:** ANOVA and chi-square analysis in the comparison between the demographic characteristics of patients classified by Fatigue Severity Scale Mean (FSS-M) categories.

Fatigue Severity Scale Mean (FSS-M)	No fatigue (≤4)	Doubtful (4.1-4.9)	Fatigue (≥5)	p-value*
**Age**	44.59 ± 11.55	48.38 ± 9.63	47.70 ± 9.16	0.033^a^
**Gender**				
Male	25 (33.3%)	3 (4.0%)	47 (62.7%)	0.058
Female	61 (20.4%)	13 (4.3%)	225 (75.3%)	
**Employment Status**				
Active	53 (35.6%)	13 (8.7%)	83 (55.7%)	<0.001^b^
Unemployed	10 (20.4%)	0 (0.0%)	39 (79.6%)	
Temporary disability	23 (14.9%)	3 (1.9%)	128 (83.1%)	
Permanent disability	0 (0.0%)	0 (0.0%)	22 (100%)	
**Previous chronic illnesses**				
No	71 (24.2%)	14 (4.8%)	208 (71.0%)	0.324
Yes	15 (18.5%)	2 (2.5%)	64 (79.0%)	
**Do you continue having symptoms?**				
No	11 (78.6%)	2 (14.3%)	1 (7.1%)	<0.001^b^
Yes	75 (20.8%)	14 (3.9%)	271 (75.3%)	
**Length of COVID-19 symptoms**				
Less than 1 month	8 (88.9%)	1 (11.1%)	0 (0.0%)	<0.001^b^
Between 1 and 3 months	7 (70.0%)	1 (10.0%)	2 (20.0%)	
Between 3 and 6 months	3 (9.4%)	3 (9.4%)	26 (81.3%)	
Between 6 months and 1 year	8 (19.0%)	2 (4.8%)	32 (76.2%)	
More than 1 year	60 (21.4%)	9 (3.2%)	212 (75.4%)	

* ANOVA for continuous outcomes and chi-square for categorical outcomes:

^a^Differences in *No fatigue (≤4) vs Fatigue (≥5), p < .05* by Bonferroni posthoc analysis for ANOVA;

^b^Differences according to chi-square for categorical outcomes *p < 0.05*

Table 3 compares the characteristics of the participants by grouping them according to the mood in which they scored the highest (eliminating ties). When comparing these groups, statistically significant differences were found between the duration (p = 0.011) and permanence of symptoms (p < 0.001), as well as employment status (p < 0.001) and mean age (p = 0.012).

**Table 3:** ANOVA and chi-square analysis in the comparison between the demographic characteristics of patients classified by Scale for Mood Assessment (EVEA) categories.

Scale for Mood Assessment (EVEA)	Sadness-depression	Anxiety	Anger-hostility	Happiness	p-value*
**(n = 122)**	**(n = 62)**	**(n = 45)**	**(n = 76)**
**Age**	49.32 ± 9.09	45.81 ± 9.53	46.53 ± 11.03	44.45 ± 10.63	0.012^a^
**Gender**					
Male	27 (44.3%)	10 (16.4%)	10 (16.4%)	14 (23.0%)	0.757
Female	95 (38.9%)	52 (21.3%)	35 (14.3%)	62 (25.4%)	
**Employment Status**					
Active	41 (33.6%)	20 (16.4%)	10 (8.2%)	51 (41.8%)	<0.001^b^
Unemployed	18 (41.9%)	12 (27.9%)	5 (11.6%)	8 (18.6%)	
Temporary disability	54 (45.4%)	27 (22.7%)	24 (20.2%)	14 (11.8%)	
Permanent disability	9 (42.9%)	3 (14.3%)	6 (28.6%)	3 (14.3%)	
**Previous chronic illnesses**					
No	99 (41.3%)	49 (20.4%)	35 (14.6%)	57 (23.8%)	0.781
Yes	23 (35.4%)	13 (20.0%)	10 (15.4%)	19 (29.2%)	
**Do you continue having symptoms?**					
No	3 (21.4%)	1 (7.1%)	0 (0.0%)	10 (71.4%)	<0.001^b^
Yes	119 (40.9%)	61 (21.0%)	45 (15.5%)	66 (22.7%)	
**Length of COVID-19 symptoms**					
Less than 1 month	2 (22.2%)	0 (0.0%)	0 (0.0%)	7 (77.8%)	0.011^b^
Between 1 and 3 months	1 (11.1%)	2 (22.2%)	1 (11.1%)	5 (55.6%)	
Between 3 and 6 months	9 (39.1%)	6 (26.1%)	5 (21.7%)	3 (13.0%)	
Between 6 months and 1 year	13 (39.4%)	10 (30.3%)	6 (18.2%)	4 (12.1%)	
More than 1 year	97 (42.0%)	44 (19.0%)	33 (14.3%)	57 (24.7%)	

* ANOVA for continuous outcomes and chi-square for categorical outcomes:

^a^Differences in *sadness-depression vs happiness, p < .05* by Bonferroni posthoc analysis for ANOVA;

^b^Differences according to chi-square for categorical outcomes *p < 0.05*

In Table 4, scores by symptom type in each of the moods on the EVEA scale showed that statistically significant differences (p < 0.001) were identified in the scores related to sadness-depression between participants who reported neurological, psychological and locomotive symptoms. In the anxiety scores the differences found in participants with psychological symptoms stand out. In the case of anger-hostility, elevated scores are related to neurological symptoms, psychological, locomotive and pain. On the contrary, the scores in happiness showed an inversely proportional behaviour and were higher in participants who did not present symptoms. When symptom types were analysed according to fatigue scores, statistically significant differences (p < 0.05) were found in all systems examined when comparing FSS scores.

**Table 4:** Score in Scale for Mood Assessment (EVEA) (sadness-depression/ anxiety/ anger-hostility/ happiness) and Fatigue Severity Scale (FSS) according to the symptomatology reported in patients.

	Scale for Mood Assessment (EVEA) sadness-depression			Scale for Mood Assessment (EVEA) anger-hostility		
Symptomatology reported	**No**	**Yes**	**p-value** ^**a**^	**No**	**Yes**	**p-value** ^**a**^
Neurological Symptoms	3.95 ± 2.65	5.20 ± 2.81	0.001***	3.15 ± 2.68	4.60 ± 2.89	<0.001***
Psychological or emotional symptoms	3.53 ± 2.47	5.96 ± 2.62	<0.001***	2.97 ± 2.50	5.26 ± 2.79	<0.001***
Locomotive Symptoms	4.25 ± 2.69	5.55 ± 2.80	<0.001***	3.44 ± 2.65	5.05 ± 2.91	<0.001***
Respiratory Symptoms	4.84 ± 3.04	5.00 ± 2.70	0.609	4.13 ± 2.84	4.40 ± 2.94	0.393
Digestive Symptoms	4.55 ± 2.81	5.29 ± 2.80	0.012*	3.82 ± 2.86	4.72 ± 2.88	0.003**
Dermatological Symptoms	4.77 ± 2.80	5.24 ± 2.84	0.127	4.16 ± 2.89	4.55 ± 2.91	0.207
Cardiac Symptoms	4.86 ± 2.88	5.10 ± 2.71	0.435	4.08 ± 2.84	4.72 ± 2.98	0.052
Kidney Symptoms	4.90 ± 2.82	5.45 ± 2.80	0.312	4.20 ± 2.88	5.47 ± 2.93	0.022*
Pain	4.25 ± 2.79	5.16 ± 2.80	0.007**	3.37 ± 2.78	4.60 ± 2.88	<0.001***
Other symptoms	5.03 ± 2.81	4.82 ± 2.81	0.477	4.37 ± 2.86	4.20 ± 2.97	0.564
Symptomatology reported	**Scale for Mood Assessment (EVEA) anxiety**			**Scale for Mood Assessment (EVEA) happiness**		
	**No**	**Yes**	**p-value** ^**a**^	**No**	**Yes**	**p-value** ^**a**^
Neurological Symptoms	3.63 ± 2.74	4.81 ± 2.87	0.001**	4.02 ± 2.73	2.81 ± 2.14	<0.001***
Psychological or emotional symptoms	3.08 ± 2.42	5.64 ± 2.72	<0.001***	3.75 ± 2.57	2.56 ± 1.99	<0.001***
Locomotive Symptoms	4.08 ± 2.81	4.99 ± 2.88	0.002**	3.38 ± 2.41	2.78 ± 2.21	0.012*
Respiratory Symptoms	4.41 ± 2.97	4.65 ± 2.84	0.431	2.71 ± 2.38	3.25 ± 2.28	0.032*
Digestive Symptoms	4.29 ± 2.86	4.81 ± 2.89	0.083	3.22 ± 2.46	2.92 ± 2.19	0.208
Dermatological Symptoms	4.49 ± 2.82	4.70 ± 2.99	0.480	3.15 ± 2.41	2.90 ± 2.18	0.309
Cardiac Symptoms	4.49 ± 2.85	4.70 ± 2.94	0.502	3.05 ± 2.37	3.07 ± 2.24	0.920
Kidney Symptoms	4.51 ± 2.89	5.16 ± 2.73	0.239	3.11 ± 2.35	2.40 ± 1.90	0.109
Pain	3.91 ± 2.90	4.77 ± 2.85	0.014*	3.47 ± 2.62	2.93 ± 2.21	0.079
Other symptoms	4.74 ± 2.86	4.31 ± 2.90	0.160	3.01 ± 2.23	3.13 ± 2.46	0.610
	**Fatigue Severity Scale (FSS)**					
Symptomatology reported	**No**	**Yes**	**p-value** ^**a**^			
Neurological Symptoms	4.83 ± 1.92	5.61 ± 1.76	<0.001***			
Psychological or emotional symptoms	5.12 ± 1.88	5.69 ± 1.74	<0.001***			
Locomotive Symptoms	4.91 ± 1.93	5.92 ± 1.57	<0.001***			
Respiratory Symptoms	4.82 ± 2.01	5.79 ± 1.61	<0.001***			
Digestive Symptoms	5.22 ± 1.84	5.65 ± 1.78	0.009**			
Dermatological Symptoms	5.25 ± 1.87	5.79 ± 1.67	0.004**			
Cardiac Symptoms	5.20 ± 1.90	5.92 ± 1.55	<0.001***			
Kidney Symptoms	5.39 ± 1.83	6.12 ± 1.48	0.016*			
Pain	4.75 ± 1.99	5.67 ± 1.71	<0.001***			
Other symptoms	5.41 ± 1.80	5.50 ± 1.85	0.632			

^a^p-value corresponding to Student’s t-test or Mann-Whitney U test according to normality test.

* *p* *<* *0.05;* ** *p* *<* *0.01;* *** *p* *<* *0.001*

**Fig 1:**
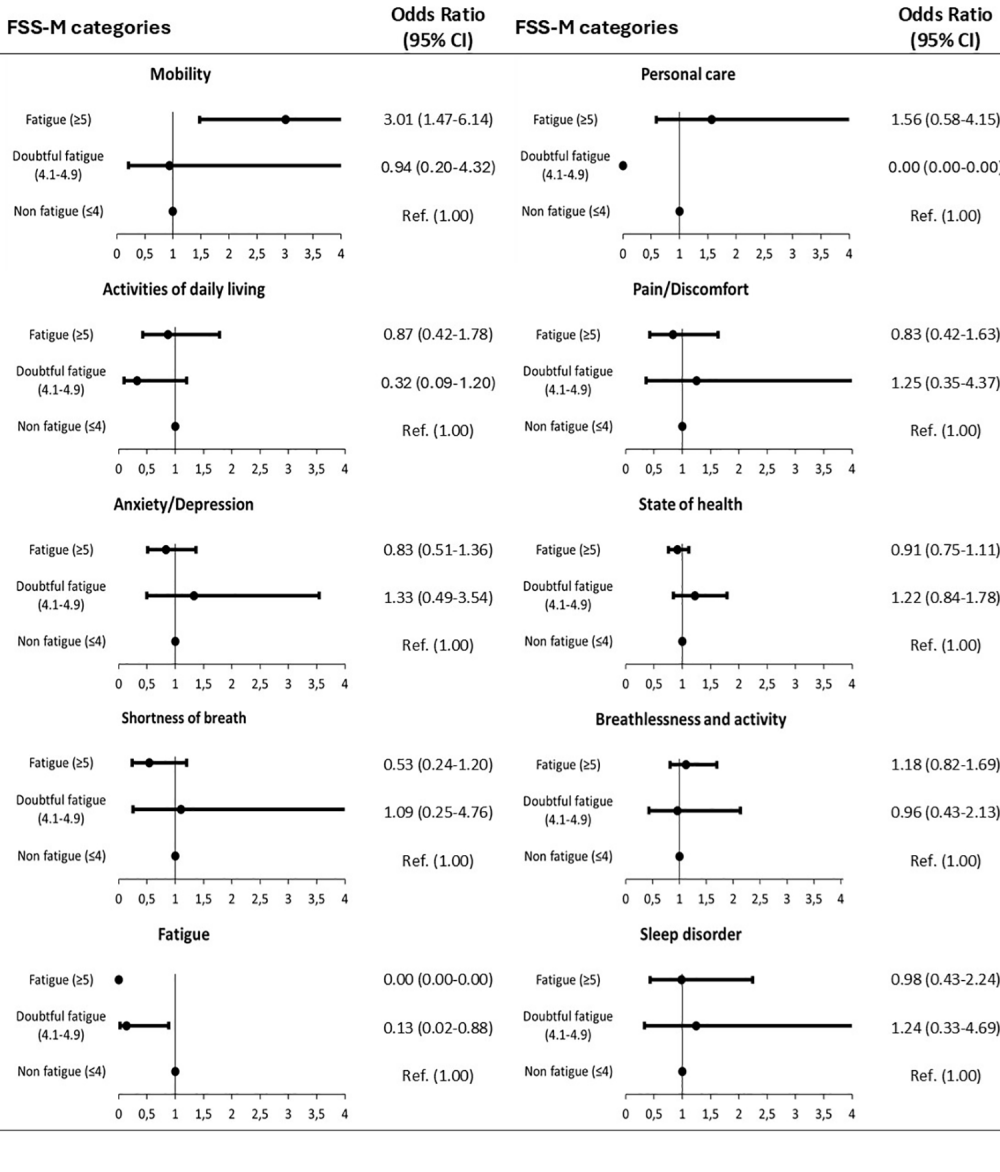
Forest plots Fatigue Severity Scale Mean (FSS-M) categories (self-perception of variables related to state of health according to FSS-M categories).

**Fig 2:**
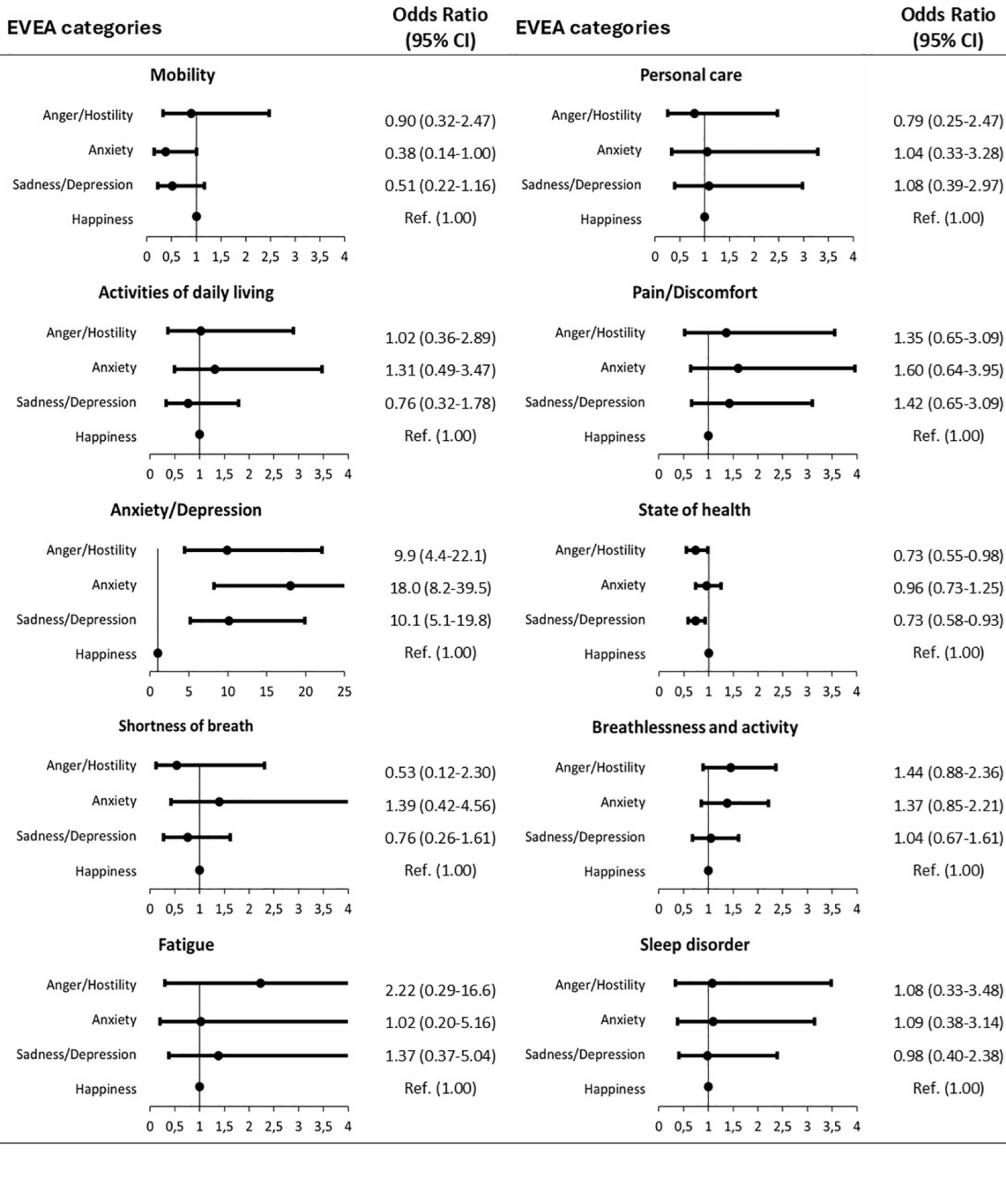
Forest plots Scale for Mood Assessment (EVEA) categories (self-perception of variables related to state of health according to EVEA categories vs happiness).

Finally, no clinically relevant relationships were found when comparing the categories of FSS (Fig 1) and EVEA (Fig 2) with the self-perception of variables related to health status.

## Discussion

This study has determined the prevalence of long-COVID symptoms in the affected Spanish population linked to associations of patients with this syndrome.

In this study the age was found to be risk factors for Long-COVID. Perlis et al. confirmed this in their study with American patients [33]. In 2023, a Polish journal published the main demographic factors related to Long-COVID. The study included female sex and advanced age, coinciding with the results of this research [34]. Consequently, this study has a sample of mostly older women as in other researches. This makes the results more easily comparable with other authors. In the study by Carvalho-Schneider et al. [35], age 40–49 years (OR 15.3, 95% CI 2.8–83.9) was found to be a significant predictor for developing Long COVID. In another study [36], bivariate analysis showed a significant association between prolonged symptoms and age 40–60 years. These findings are consistent with the results of this study, as the mean age of patients with symptoms was 47 years. In our study it is worth underlining the high number of patients who continued with symptoms after 12 months of their onset, 75.1%, compared to results obtained by Perlis et al. [33], who collected up to 15.2% of participants reporting any symptoms. Although female gender and age have been part of clinical trials and meta-analyses, the need for clinical trials that classify patient groups according to these variables to make better decisions on prevention, diagnosis and management of the disease is emphasized [37].

Our study found that the most frequent symptoms were fatigue, neurological symptoms, pain, respiratory disorders, and psychological problems, in that order. Fatigue is unquestionably a very common symptom in Long-COVID. The Office for National Statistics (ONS) cohort study in the United Kingdom, which included control participants, reported that fatigue was present in 8.3% of cases [38]. In our results, the percentage of fatigue was present in 72.7% of cases. Likewise, in their study, Percze et al. indicated that fatigue was present in 70% of patients [39]. Similar results have been found by other authors [40]. A meta-analysis revealed that the proportion of people experiencing fatigue 12 or more weeks after COVID-19 diagnosis was 0.32 (95% CI, 0.27, 0.37; p < 0.001; n = 25,268; I 2 = 99.1%) [15]. The pathogenesis of fatigue involves several reasons, including inflammatory, neurological, metabolic, muscular, psychological, and social factors. Chronic fatigue syndrome is known as sequelae in some acute infections, which suggests that this may be the case in Long-COVID. Regarding the severity of fatigue assessed by the FSS scale, in a study carried out in several Arab countries, high scores were obtained in patients who had suffered from COVID-19 [41].

Some of the articles reviewed describe mental and cognitive symptoms, such as confusion and difficulty concentrating, which were also found in our study [17,42,43]. Guziejko et al. describe that Long-COVID presents neurological symptoms such as cognitive impairment, brain fog, loss of concentration or memory problems, headaches, sleeping disorders and mobility problems. In addition, patients generally showed poor ability to concentrate [44]. The proportion of people who had cognitive impairment in a meta-analysis was 0.22 (95% CI, 0.17, 0.28; p < 0.001; n = 13,232; I 2 = 98.0) [15]. A study published by Ariza et al. in 2023 showed that the severity of symptoms during COVID-19 infection influenced the presence of poor executive function in patients who developed Long COVID [45]. It seems clear that neurological symptoms in Long COVID patients are related to fatigue and mood disorders.

It is found in this study that pain is a common symptom in Long COVID patients and that it is related to both fatigue and emotional state. Among the neurological symptoms, headache stands out, although with very scattered estimates (ranging from 8% to 44%). In addition, studies show that about a quarter of patients with persistent symptomatology report concentration difficulties. Memory loss is also described in all systematic reviews, although with scattered estimates (between 16% and 18%) [37,38,46,13]. A review of the literature indicated that long-COVID patients were increasingly reporting musculoskeletal pain as one of the most common persistent symptoms. However, the pathophysiological mechanisms that produce it, as well as the characteristics of this type of pain, are unknown [47]. It is worth noting that the NICE Persistent COVID Quick Guide lists muscle and joint pain among the common symptoms, demonstrating that this symptom is relevant [48].

A meta-analysis indicated that up to 21% of long-COVID patients had dyspnoea. It also showed heterogeneity in the presence of symptoms depending on the patient’s environment and their healthcare system [49].

The symptoms of the long COVID patients in this research are more frequently prolonged in those suffering from fatigue or psychological disturbances. Another review of the literature shows that although psychological symptoms were not the most prevalent overall, they tended to appear in long-COVID patients in the form of anxiety, depression, and post-traumatic stress syndrome [21].

Although the use of the EVEA scale for the assessment of mood in Long-COVID is widely recommended, articles including it are scarce. In any case, what seems clear is that the disease affects the mood of patients in a relevant way [41]. The proportion of people reporting psychological symptomatology, mainly in the form of anxiety (between 18% and 29%) and depression (between 15% and 20%) is notable [50]. Considering the influence of some variables on mood, it is worth noting that age was related to levels of sadness and happiness in this study, suggesting that factors such as the patient’s stage of life may influence mood. Morin et al. found that patients aged 75 and older were the most affected [51]. A literature review study showed that the frequency of severe depressive symptoms ranged from 3 to 12%. The severity of acute-phase COVID-19 was not associated with the frequency of depressive symptoms [52].

Whether or not they were working, as well as the length of time the patient had been symptomatic, was connected to all mood subscales (sadness-depression, anxiety, anger-hostility, and happiness). This reveals that factors such as the situation at work and the chronicity of the symptoms could comprehensively affect the person’s emotional well-being. An international cohort study indicated that 45.2% of Long-COVID patients required reduced work hours compared to pre-illness and that 22.3% were not working at seven months from symptom onset [24]. Furthermore, it was shown that disability laws could lead to job loss and involuntary retirement for said vulnerable workers, which in turn was linked to poorer emotional health [53]. According to the results of this and other studies, the disease leads to absences from work [24,53].

Sadness was associated with a wide range of symptoms in this research. Other research showed that as time passed and symptoms reduced, the emotional state improved in these patients. This result is in line with the articles by Fancourt et al. and Bota et al. [19,54].

Anxiety was associated with physical symptoms in this research. This finding supports the notion that anxiety disorders have both psychological and somatic manifestations. In a qualitative study, it was found that seeking reassurance and knowledge, developing greater self-awareness, and building pleasure and comfort were coping strategies for daily life in the face of the symptoms and dysfunction that this syndrome entails. Reassurance is the opposite of anxiety, so effective coping strategies to help manage anxiety should be enhanced in these patients [55].

It was also found that anger and hostility were related to other symptoms. This demonstrates that negative emotional states can have repercussions on multiple body systems [56]. On the other hand, happiness was associated with physical and psychological symptoms, suggesting that positive moods may have beneficial effects on a physical and mental level [57].

Taken together, these findings highlight the close relationship between mood and a wide variety of symptoms, underscoring the importance of addressing emotional factors in comprehensive health management. In addition, those who suffered from depression prior to infection tend to be the ones who most often show long-term sadness [58]. Health professionals should carefully consider the assessment and treatment of mood as a fundamental part of these patients’ healthcare and even more so in those who have previous depressive pathology, as it is a clear risk factor for poorer mental health. In order to avoid chronicity, it would be advisable to treat depressive states with SSRIs, since the study by Mazza et al., found that the response of patients with post-COVID syndrome is rapid with these drugs [59]. Brain fog and sensory overload decreased the most, followed by chronic fatigue [60].

The fatigue presented by the patients also had an impact on their work environment as well as their mood, as in some cases they were unable to carry out the work prior to infection due to health disorders that arise. Kerksieck et al. estimated that the proportion of patients with post-COVID syndrome who did not regain full work capacity after 1 year was 5.8% [61].

It should therefore be noted that fatigue and mood alterations generate an incapacity for work in these patients and, associated with this, a loss of years of life due to disability. This leads to in a higher healthcare cost, not only because of the consultations and drugs required, but also owing to the loss of work assets at an early age. This issue warrants further investigation of this syndrome in order to avoid especially long-term symptoms and consequently disability [62,63].

On a practical level, specific programmes should be carried out with these patients to improve their emotional state and reduce the level of fatigue. Among the actions that could be carried out are: energy management techniques (pacing), establishing routines that help to structure a balanced schedule, encouraging light and adapted physical activity, using relaxation techniques, creating safe spaces where the patient can express themselves without being judged, engaging in pleasurable activities, adequate hydration and nutrition, sleep hygiene, creating support groups for patients and families, as well as close and regular monitoring of the patient [64,65].

### Limitations

There are, however, certain limitations to our study that must be considered. Firstly, we have used non-random sampling, although as the sample volume has been exceeded, any bias may have been controlled. Even so, it may be the case that symptomatic patients are more represented by the selective inclusion effect. The second is that the signs and symptoms were self-reported, but previous studies indicate a high accuracy in the statements of long-COVID patients and therefore there would be no loss of sensitivity in this research. However, chronobiological aspects have not been taken into account to assess fatigue and mood as Adan proposed [66,67]. The third limitation lies in the transversality of the design, which curbs the search for causality and knowledge of the incidence of the syndrome. The last limitation is due to the fact that the study is carried out exclusively in the Spanish population. Because of this, the results cannot be extrapolated to populations in other social contexts.

### Strengths

As for its strengths, this study is one of the first in Spain not to focus exclusively on patients who have been hospitalised, but to include any long-COVID patient belonging to an association of people affected with this syndrome. This allows us to garner a global vision of the convalescence associated with the syndrome. In addition, validated scales for mood and fatigue have been used to ensure the reliability of the data.

### Areas of research to be explored

Future lines of research should thoroughly explore how these patients experience their levels of fatigue to further understand the problem through qualitative phenomenological studies. With this, it would be possible to propose actions for improvement in the long-COVID patient follow-up programs that focus on individuals and their experiences. It will be interesting to incorporate a quality-of-life evaluation with validated instruments and related it with fatigue and mood in these patients.

## Conclusions

This study highlights the significant impact of long COVID-19 on physical and emotional health, with fatigue and psychological disorders being common. Older people are the most affected by persistent symptoms. Mood disorders, such as anxiety and depression, are closely related to physical symptoms, highlighting the need for holistic healthcare approaches.

## Supporting information

S1 File STROBE_checklist_cohort. (DOCX)

S1 Data Long COVID (english version). (XLSX)
